# Disease of the Lungs

**Published:** 1901-03-16

**Authors:** 


### DISEASE OF THE LUNGS.

GORDON[1] writes of the relation of wind to the prevalence of phthisis in Devonshire. He finds that the death-rate from phthisis is most pronounced where the west and south-west winds have most access in the district. He compares numerous rural districts with one another, according to their configuration and their wind prevalence, and finds that Tavistock is the most exposed to the west and south-west, and shows the maximum phthisis mortality. Axminster, however, with the lowest death-rate from phthisis, has a most wind-protected formation. He shows, however, that such large units of area as rural sanitary districts are disadvantageous on account of the difficulty in comparison with natural features, the same district being part exposed and part sheltered.

He therefore divides his districts into groups and compares these, and again comes to the conclusion that where there is most exposure to the west and south-west winds, there exists the highest death-rate per thousand from phthisis. He also adduces evidence to show that the nature of geological formations is subordinate to the exposure to wind as to its influence on the death-rate of phthisis.

Emile Boix[2] calls attention to the atrophy of the muscles of the shoulder as an early sign of phthisis. Together with the habitual tenderness, this muscular atrophy is an early but not a primary sign. It is to be most marked by palpation of| the deltoid supra and intraspinatus, as well as the appearance of the supra and subclavicular forsæ.

Thomas Harris[3] reports that he found 38·84 per cent. of the post-mortem examinations made on all cases of disease or accident over twenty years of age, presented signs to the naked eye of obsolete or quiescent tubercle. Of 192 cases of his own, after allowing at least five years to intervene, he traced 85, of whom 56 were dead, 9 invalids, and 20 are in good health. These latter 20 were found to have been noted as cases where the physical signs were present over a very limited area. This was before the open-air treatment came into repute. With regard to the open-air treatment, he refers to the freedom from phthisis found among the nomad tribes, and the susceptibility of the tribes building stone houses. But statistical comparisons between typical town and country districts show that for the younger persons the mortality is greater in the rural districts, while at a more adult age the town mortality is greater. He suggests common lodging houses as sources of infection in towns. He urges the formation of special sanatoria as a protection to the community, as an educationary movement, and as a means of cure, though he warns that the cure may not be absolute for a long time, even after it is apparently certain.

Rube[4] quotes Weicker's sanatorial figures. The cases were classified in three categories—

Class 1.—The initial state.
„ 2.—Past the initial state.
„ 3.—Advanced phthisis with destructive process.

Of 1,367 patients who were discharged up to January, 1899, only 8 per cent. failed to reply to inquiry cards. Of those who answered 95 per cent. were still working who were discharged in 1898, 97 per cent. of those discharged in 1897, and 100 per cent. of those discharged in 1896. But of those in the third state 57 per cent. were dead of the 1898 series, 80 per cent. were dead of the 1897 series, 94 per cent. were dead of the 1896 series. He also states that the Hanover State Insurance Institutes have come to the conclusion that their interests are best served in "instituting an early and thorough treatment of all their sick." The expenses incurred in treatment being saved in pensions which would otherwise have been paid.

Maguire[5] in his Harveian lectures describes his views of the prognosis and treatment of pulmonary tuberculosis. He mentions in his first lecture a "multiple pleuritic" onset in which the patient shows little ill-health, but scattered pleuritic rubs may be heard by the medical man, and the course of the case is rapid and grave. Emphysematous phthisis (of Mitchell Bruce) with choking cough and bronchitic sputum containing tubercle bacilli, and yet only the physical signs of emphysema, is a serious form. A bad prognosis should be entertained "if the symptoms are out of all proportion greater than the physical signs." Some tuberculous patients are of florid, full habit, and they show a worse course than the pale anæmic ones. The prognosis is bad in cases of deformity in the chest; but where the onset is secondary to infected lymph glands the course may be slower. The removal of such glands aids the beneficial result. Pleuritic effusion may be beneficial when occurring in the course of the disease, and should be left alone; but empyema is of grave import.

He further[6] discusses the prognosis under the heads of the symptoms—pyrexia, general weakness, digestive disturbances, and hæmoptysis. Absence of pyrexia may mean a harmful absence of resistance by the body to the poison of the bacteria. But high pyrexia may be due to neurosis, or be the onset of an ordinary attack. But generally speaking it is a bad factor in prognosis. A morning temperature higher than the evening is bad (Kingston Fowler). Cardiac and vaso-motor weakness, digestive disturbances early in case, hæmoptysis, if copious and frequent, one large hæmoptysis with pyrexia if the diagnosis is ascertained, are all bad factors in the early stage. In the second stage large ("echoing") rales mean softening of inflammatory tissues and are an adverse sign.

Spread of infection takes place by (*a*) simple contiguity of tissue; (*b*) lymphatic absorption; (*c*) bronchial insufflation; (*d*) venous conduction; (*e*) arterial conduction. The wider spread, especially if another lobe is attacked (usually by lymphatic absorption); the detection of an isolated patch in another lobe, due to insufflation, and blood infection are all grave factors.

Infection of larynx or intestinal tract, hectic pyrexia and hæmoptysis (in second stage) are bad. Dyspnœa in third stage due to fibrosis is more favourable than that due to cavitation. Failure of circulation, the sudden production of pneumothorax hæmoptysis, and are all dangerous in this stage. Any case, if cured, may relapse.

He then discusses[7] the treatment of pulmonary tuberculosis, and treats his subject under four heads, viz.:—(1) How to increase the normal resistance of the tissues to the invasion of the bacillus and its congeners; (2) how to hinder the action of the bacillus by destroying its congeners the staphylococcus pyogenes and the pneumococcus; (3) how to hinder the action of the b. tuberculosis by interfering somewhat with its own vitality; and (4) how to attempt to kill the b. tuberculosis *in situ*. With regard to the first, the open-air treatment is useful if used with common sense. As to drugs, arsenic is very valuable and sodium cacodylate is a useful form of this, as it can be given in larger doses than other forms. Various methods have been used to increase the phagocytic powers of the leucocytes, viz., glycogen (Habershon), yeast (De Backer), nuclein (Maguire). He tried injections of takadiastase solutions (in formic aldehyde) into the venous circulation, with the idea of inducing the mixture of it in the blood, on passing through the lungs, to dissolve the cellulose coats of the bacteria. There was a marked febrile reaction. He used "liquor nucleinicus" also intravenously, also with marked febrile reaction. He then tried formic aldehyde alone in strengths of 1 in 250,000 to 1 in 2,000, from a specially-made "burette-pump." Injecting some "2 cc. in 5 heart-beats," he could inject 50 cc. daily, and the result is encouraging. Nearly all of 70 cases showed some improvement. A case of "multiple pleuritic" pulmonary tuberculosis is practically well, and many others have lost all the tubercle bacilli from the sputa. A case of putrid bronchiectasis was treated thus satisfactorily. Stronger solutions can be used, but there is a danger of albuminuria, hæmaturia and thrombosis.

[1] Brit. Med. Jour., Jan. 12, 1901. [2] Lancet, Dec. 15, 1900. [3] Med. Chron., Nov. [4] Tuberculosis, Jan. 1901. [5] Lancet, Dec. 1. [6] Lancet, Dec. 8. [7] Brit. Med. Jour., Dec. 15, 1900.

